# Supplementary material for: Structural and functional characterization of the interaction between the influenza A virus RNA polymerase and the CTD of host RNA polymerase II
Source: J Virol. 2024 Apr 2;98(5):e00138-24. doi: 10.1128/jvi.00138-24 (PMC11092357; doi:10.1128/jvi.00138-24)
Supplement: Supplemental tables and figures — Tables S1 and S2; Figures S1 to S6. [file jvi.00138-24-s0001.pdf]

SUPPLEMENTAL MATERIAL

Structural and functional characterisation of the interaction between the influenza A virus RNA polymerase and the CTD of host RNA Polymerase II

Jeremy Keown,[a]* Alaa Baazaoui,[b] Marek Šebesta,[c] Richard Štefl,[c,d] Loic Carrique,[a] Ervin Fodor,[b]# Jonathan Grimes[a]#

[a]Division of Structural Biology, Wellcome Centre for Human Genetics, University of Oxford, Oxford, United Kingdom
[b]Sir William Dunn School of Pathology, University of Oxford, Oxford, United Kingdom
[c]CEITEC–Central European Institute of Technology, Masaryk University, Brno, Czechia
[d]National Centre for Biomolecular Research, Faculty of Science, Masaryk University, Brno, Czechia

Running Head: Interaction of the influenza polymerase with Pol II

#Address correspondence to Ervin Fodor, ervin.fodor@path.ox.ac.uk; Jonathan Grimes jonathan.grimes@strubi.ox.ac.uk.

*Present address: School of Life Sciences, University of Warwick, Coventry, United Kingdom

Jeremy Keown and Alaa Baazaoui contributed equally to this work. Author order was determined on the basis of seniority.

Peptide	Schematic and sequence
pS2	 Y(pS)PTSPS Y(pS)PTSPS Y(pS)PTSPS Y(pS)PTSPS
pS5	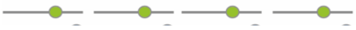<br>YSPT(pS)PS YSPT(pS)PS YSPT(pS)PS YSPT(pS)PS
pS7	 YSPTSP(pS) YSPTSP(pS) YSPTSP(pS) YSPTSP(pS)
pS(2/5).1*	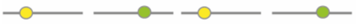<br>Y(pS)PTSPS YSPT(pS)PS Y(pS)PTSPS YSPT(pS)PS
pS(2/5).2*	 YSPT(pS)PS Y(pS)PTSPS YSPT(pS)PS Y(pS)PTSPS
pS(5/7).1*	 YSPT(pS)PS YSPTSP(pS) YSPT(pS)PS YSPTSP(pS)
pS(5/7).2*	 YSPTSP(pS) YSPT(pS)PS YSPT(pS)PS YSPT(pS)PS
pS2 pS5**	 Y(pS)PT(pS)PS Y(pS)PT(pS)PS Y(pS)PT(pS)PS Y(pS)PT(pS)PS
pS5 pS7**	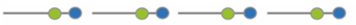<br>YSPT(pS)P(pS) YSPT(pS)P(pS) YSPT(pS)P(pS) YSPT(pS)P(pS)
Scrambled (control peptide)	PSSSTPSSYTPSPSSSPTSYSPYYTSPP

TABLE S1 Design of Pol II CTD mimic peptides with different phosphorylation states and simplified schematic to depict phosphorserines (pS) shown as coloured circles (pS2 (yellow), pS5 (green), pS7 (blue)). *Alternating phosphorylation of serine residues per heptad repeat. **Double phosphorylation of serine residues per heptad repeat.

	1918 vRNA 4rpt peptide<br>EMD-18945<br>PDB-8R60	1918 vRNA 4rpt peptide Ordered PB2<br>EMD-18947<br>PDB-8R65
**Data collection**		
Microscope	Titan Krios G3i (OPIC)	
Voltage (kV)	300	
Detector	Gatan K2 with EF	
Recording mode	Counting	
Magnification	130,000	
Movie/micrograph pixel size (Å)	1.05	
Dose rate (e-/px/sec)	9.6	
Number of frames per movie	40	
Movie exposure time (s)	4.2	
Total dose (e-/Å^2)	40	
Defocus range (um)	-1.2 to -2.5	
**EM data processing**		
Number of movies/micrographs	7,921	
Box size (px)	256	
Particle number (total)	4,231,383	
Particle number (used in final map)	303,608	16,121
Symmetry	C1	
Map resolution (Å, FSC 0.143)	3.23	4.23
Map sharpening B-factor (Å^2)	167.9	133.5
**Model Building and Validation**		**Rigid-body refinement only**
Initial model used	7NHX	7NHX/8R60
Model composition		
Non-hydrogen protein atoms	27,951	35,542
Protein residues	1707	2196
Ligands	23	23
B factors (Å^2) - mean		
Protein	77	317
Nucleotide	59	169
RMSD from ideal		
Bond length (Å)	0.004	0.005
Bond angles (°)	0.501	0.621
Validation		
Molprobity score	2.01	2.23
Clashscore	8.1	11.5
FSC (0.5) model-vs-map	3.5	7.6
CC model-vs-map (masked)	0.79	0.66
Ramachandran plot		
Favored (%)	93.9	92.8
Allowed (%)	6	6.7
Outliers (%)	0.1	0.5

TABLE S2 Cryo-EM data collection and model refinement statistics.

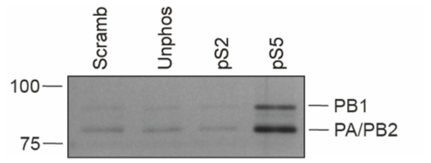

FIG S1 Effect of serine phosphorylations of Pol II CTD on binding the 1918 pandemic H1N1 influenza A virus polymerase. Controls for the Pol II CTD binding assay, including a scrambled (Scramb), unphosphorylated (Unphos), serine 2 phosphorylated (pS2), and serine 5 phosphorylated (pS5) Pol II CTD mimic peptides.

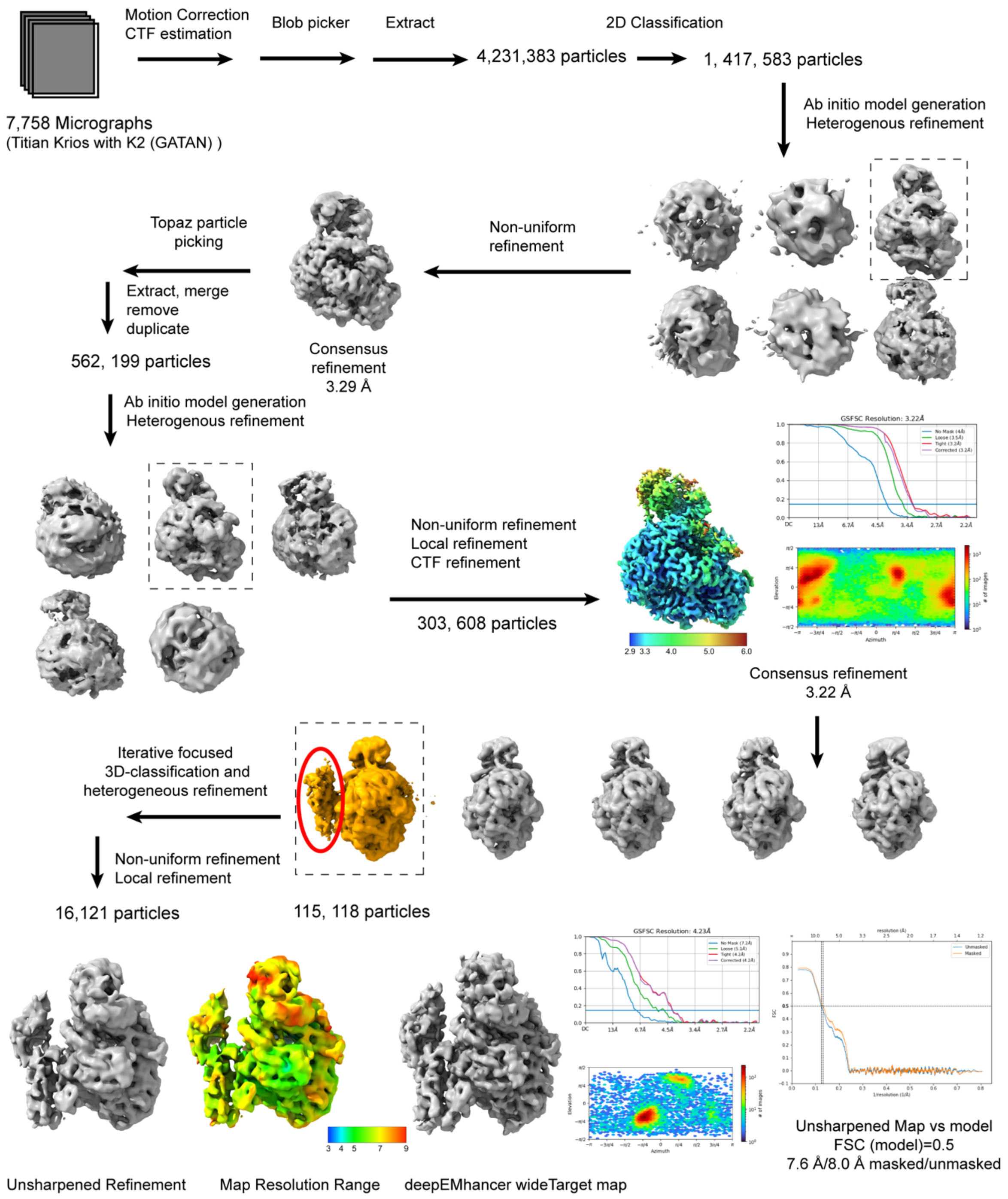

FIG S2 Cryo-EM processing scheme.

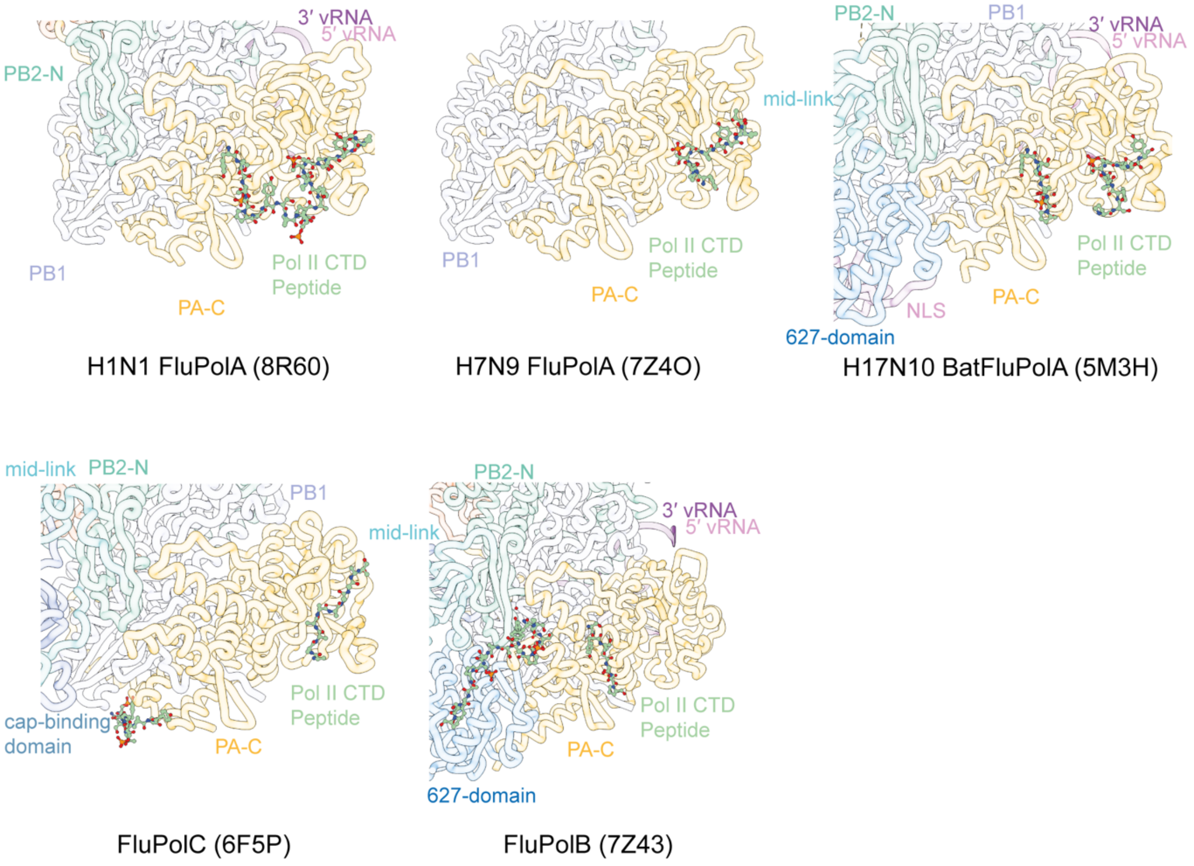

FIG S3 Comparison of the CTD binding site of influenza virus polymerases of different influenza virus types with all models shown in the same orientation.

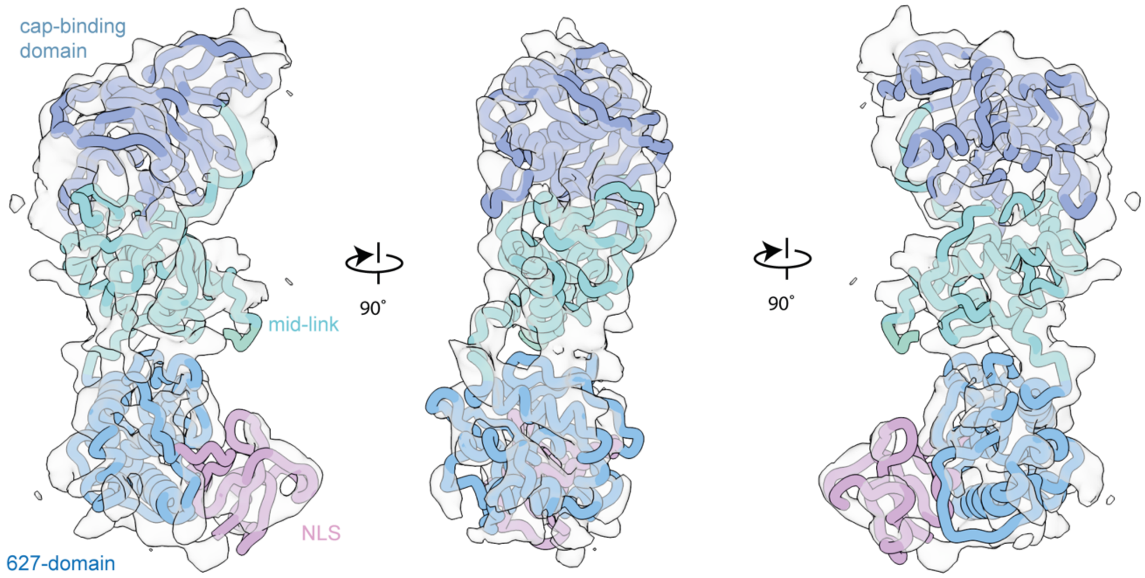

FIG S4 DeepEMhancer map showing the fit of the PB2-C domains from the new PB2 conformation. Model shown is PBD ID 8R65.

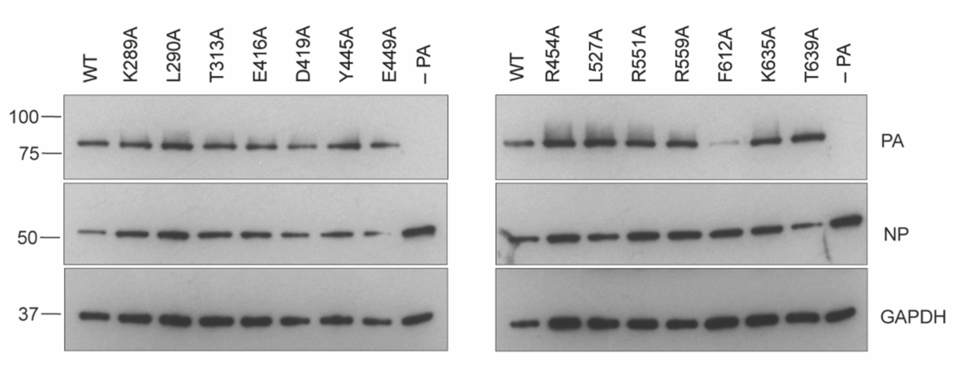

FIG S5 Expression of the PA (WT and mutants) and NP of the 1918 pandemic H1N1 influenza A virus polymerase. HEK293T cells were transfected to express PB1, PB2 and PA (WT or mutant) polymerase subunits, NP, and segment 6 vRNA and cell lysates were analysed by SDS-PAGE and Western blotting with antibodies against PA, NP and GAPDH (loading control). A representative blot with size markers (kDa) is shown.

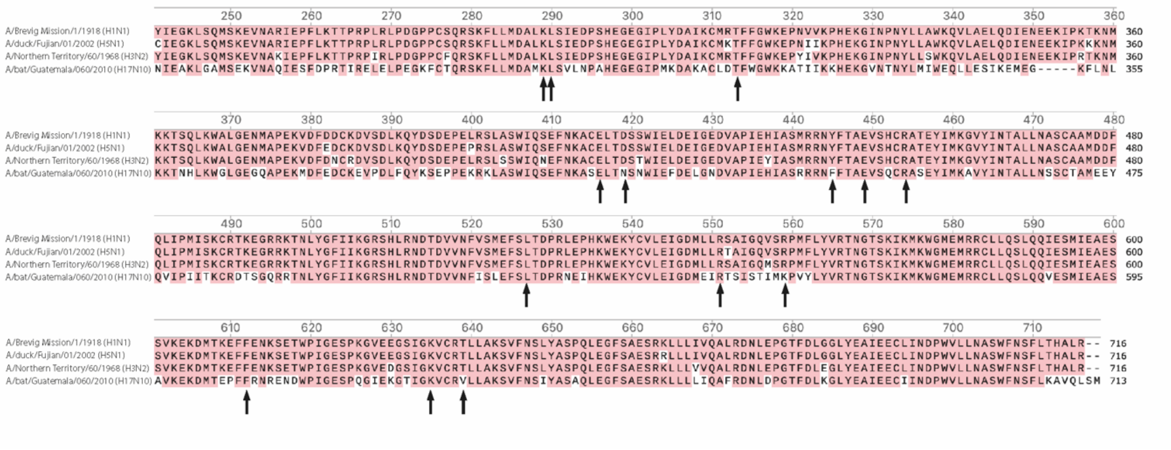

FIG S6 Identification of conserved residues in the CTD-binding site of the influenza A virus PA subunit. Multiple sequence alignments of PA-C from A/Brevig Mission/1/1918 (H1N1) (UniProt Q3HM39), A/duck/Fujian/01/2002 (H5N1) (D3YNB8), A/Northern Territory/60/1968 (H3N2) (P03434), and A/bat/Guatemala/060/2010 (H17N10) (H6QM92) using Clustal-Omega in SnapGene. Identical residues are coloured in red. Black arrows depict conserved residues that were hypothesised to affect CTD binding based on our structural model of pS5 CTD-bound 1918 pandemic H1N1 influenza A virus polymerase.
